# Nontuberculous Mycobacterial Flexor Tenosynovitis of the Wrist and Hand

**DOI:** 10.7759/cureus.58716

**Published:** 2024-04-22

**Authors:** Tatevik Malisetyan, Skylar R Harmon, Mariafe Reyes, Mohammadali M Shoja, Gary Schwartz

**Affiliations:** 1 Department of Medical Education, Nova Southeastern University Dr. Kiran C. Patel College of Allopathic Medicine, Fort Lauderdale, USA

**Keywords:** tenosynovitis, nontuberculous mycobacteria, musculoskeletal infections, immunocompromised, hand infection

## Abstract

Nontuberculous mycobacteria (NTM) are uncommon causes of cutaneous and musculoskeletal infections. Here, we present an immunocompromised patient with persistent swelling in the left hand, wrist, and distal forearm. MRI findings revealed flexor tenosynovitis with synovial hypertrophy of the left hand and wrist and loculated fluid containing rice bodies along the distal flexor digitorum muscles in the volar aspect of the left wrist. The patient underwent flexor tenosynovectomy, and histological examination of the excised tenosynovium and mass revealed noncaseating granulomas. *Mycobacterium intracellulare* was identified in microbiological cultures. Antimycobacterial therapy was administered postoperatively to manage the infection. This report underscores the significance of maintaining a high index of suspicion for NTM infection when assessing chronic hand swelling, particularly in individuals with compromised immune systems.

## Introduction

Nontuberculous mycobacteria (NTM) represent a diverse group of bacteria commonly found in soil, swamps, and water sources [1]. Infections with NTM most frequently occur in the lungs, particularly in individuals with pre-existing lung conditions, although approximately 10-15% of cases involve extrapulmonary sites [1-3]. Extrapulmonary infections, while rare, can affect the skin and, less commonly, the musculoskeletal system [3,4]. When musculoskeletal involvement occurs, the hands and wrists are often affected, typically presenting as granulomatous tenosynovitis [5,6]. NTM infections are primarily transmitted through direct inoculation, but in immunocompromised individuals, they can result from disseminated pulmonary disease [7]. *Mycobacterium marinum*, *Mycobacterium kansasii*, and *Mycobacterium avium* complex are among the most frequently identified NTM species, with their prevalence varying depending on geographic location [8].

Optimal management of NTM tenosynovitis typically involves a combination of surgical excision and prolonged antimycobacterial therapy [9]. However, delayed diagnosis can lead to treatment failure and further disease extension into deeper tissues, resulting in significant morbidity. Timely diagnosis is therefore critical for achieving favorable outcomes [10]. Diagnosis is established through microbiological culture from synovial tissue or fluid [11]. NTM species are challenging to isolate on culture, and initial negative results should not delay treatment if clinical suspicion is high [12]. A comprehensive approach, including detailed clinical assessment, radiographic analysis, and pathological evaluation, alongside a thorough exploration of the patient's medical history, is essential for the early detection and treatment of the infection. Here, we present a case of NTM infection affecting the wrist and hand in an immunocompromised individual, resulting in hand flexor tenosynovitis.

## Case presentation

A 45-year-old right-hand dominant male, with a medical history significant for hypertension, hepatitis A, and human immunodeficiency virus (HIV) infection, presented to our orthopedic clinic with persistent swelling in the left small finger, left wrist, and distal forearm lasting approximately one and a half years. Additionally, he reported the development of a nodule on the palmar aspect of the left hand, which had become increasingly noticeable over the past three months. Despite an initial evaluation by another healthcare provider one year ago, who recommended X-ray imaging, the patient missed subsequent follow-up and imaging appointments. The patient denied any history of traumatic injury, similar previous problems, or associated discomfort. At the presentation, he was receiving antiretroviral agents, including abacavir/lamivudine and efavirenz, for the management of HIV infection. Systemic symptoms such as fever or weight loss, as well as pulmonary symptoms such as cough, were absent.

**Figure 1:**
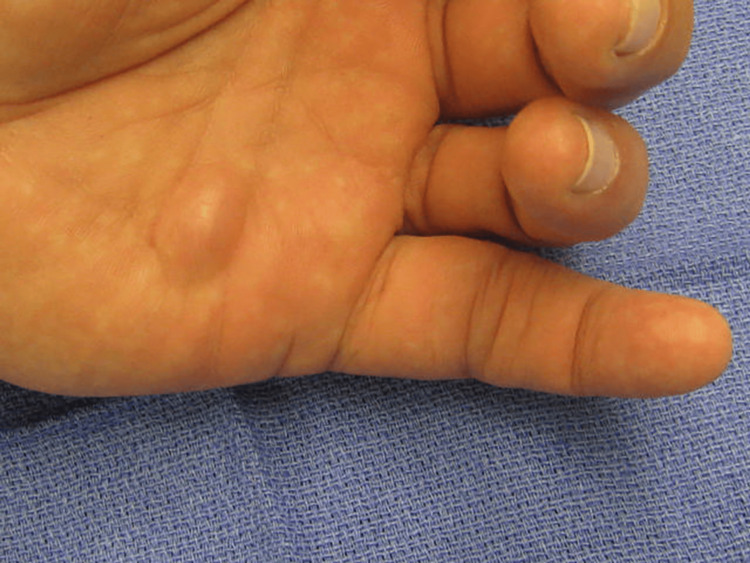
Preoperative clinical findings A conspicuous swelling is seen in the volar aspect of the hand between the distal and proximal palmar creases

A physical examination of the left upper extremity revealed a normal range of motion of the shoulder, elbow, and forearm. The left wrist exhibited the following range of motion: dorsiflexion 0-50 degrees (normal range: 0-60 degrees), volar flexion 0-50 degrees (normal range: 0-60 degrees), radial deviation 0-15 degrees (normal range: 0-20 degrees), and ulnar deviation 0-20 degrees (normal range: 0-30 degrees). He had limited range of motion in the left small finger, with specific findings including active range of motion of the metacarpophalangeal (MCP) joint at 0-50 degrees (normal: 0-90 degrees), proximal interphalangeal (PIP) joint at 10-60 degrees (normal: 0-90 degrees), and distal interphalangeal (DIP) joint at 0-40 degrees (normal: 0-80 degrees). The small finger came within 4.0 centimeters of the distal palmar crease. Examination of the volar aspect of the proximal and middle phalanges revealed bogginess extending proximally to the proximal palmar crease, with a soft tissue mass noted between the proximal and distal palmar creases, possibly contiguous with the small finger. Swelling and bogginess were also observed on the volar ulnar aspect of the left wrist, with no erythema noted and normal capillary refill and skin turgor (Figure 1).

**Figure 2:**
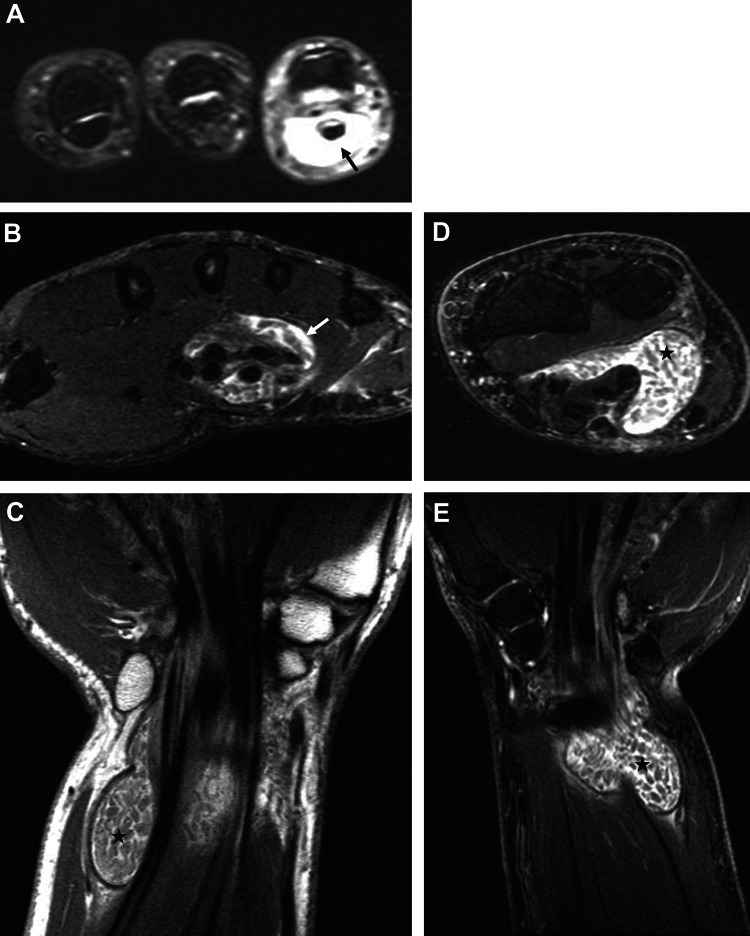
Preoperative MRI of the left hand, wrist, and distal forearm In the axial image of the fingers (A), flexor tenosynovitis of the left small finger is highlighted by a black arrow. Moving to the axial image of the hand at the level of metacarpal bones (B), both flexor tenosynovitis and synovial hypertrophy are evident, indicated by a white arrow. The axial (D) and coronal (C and E) images of the left wrist and distal forearm show a loculated fluid in the volar aspect of the wrist, marked by black asterisks, containing rice bodies

Radiographs of the left small finger and wrist revealed no fractures, dislocations, foreign bodies, lytic or blastic lesions, or carpal malalignment. An MRI of the left hand, fingers, wrist, and distal forearm demonstrated flexor tenosynovitis with synovial hypertrophy and a 4.3 cm area of loculated fluid along the distal flexor digitorum muscles in the volar aspect of the left wrist, containing rice bodies (Figure 2).

**Figure 3:**
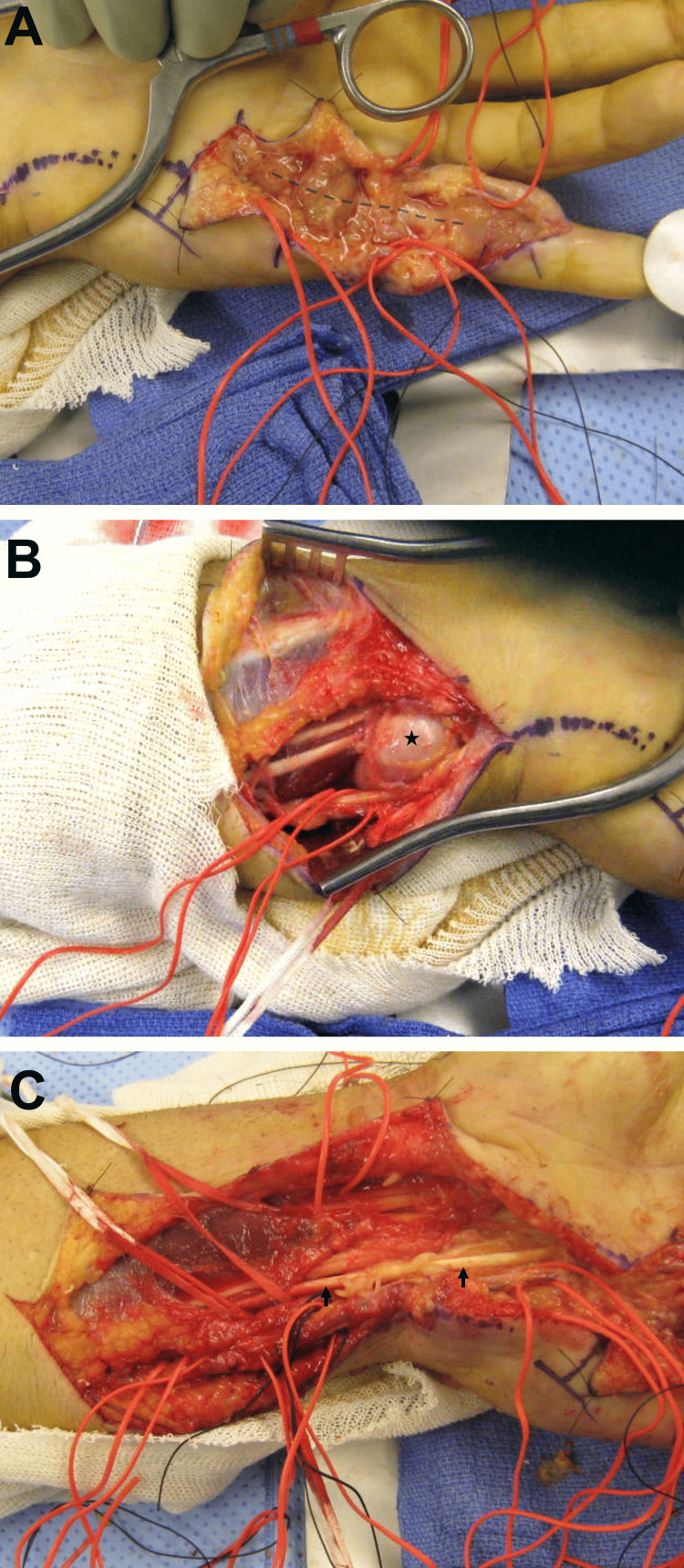
Intraoperative findings Intraoperative findings of the (A) left small finger showing flexor tenosynovitis (tenosynovium is marked by the dashed line), (B) volar ulnar aspect of the left wrist, showing a soft tissue mass (black asterisk), and (C) left distal forearm and wrist, demonstrating the flexor tendons (black arrows) after the flexor tenosynovectomy is performed

The patient underwent flexor tenosynovectomy of the left small finger, hand, wrist, and forearm, along with a carpal tunnel release (Figure 3). The tenosynovitis was non-purulent and did not exhibit a foul odor.

Specimens from the left small finger tenosynovium and mass from the left distal forearm were sent for pathologic and microbiologic evaluation. The histopathological examination revealed partially necrotic synovial tissue with noncaseating granuloma, inflammation, and reactive changes with collagenized connective tissue and fibrinous inflammatory debris. The culture grew acid-fast bacilli, which was identified as a *Mycobacterium intracellulare* via polymerase chain reaction-restriction fragment length polymorphism analysis (PRA) by the Department of Health.

The patient received a six-month course of clarithromycin and ethambutol postoperatively. He underwent occupational hand therapy for four months before transitioning to a home therapy program. At a follow-up appointment four months after surgery, all incisions were healed. The active range of motion of the wrist measured 0-50 degrees in both dorsiflexion and volar flexion (normal range: 0-60 degrees). In the left small finger, the MCP joint exhibited a range of motion of 0-85 degrees (normal: 0-90 degrees), the PIP joint showed a range of motion of 0-60 degrees (normal: 0-90 degrees), and the DIP joint had a range of motion of 0-40 degrees (normal: 0-60 degrees) (Table 1).

**Table 1:** Preoperative and postoperative active ROM for the left wrist and small finger joints ROM: range of motion

	Preoperative ROM	Postoperative ROM	Normal ROM
Dorsiflexion, wrist	0-50 degrees	0-50 degrees	0-60 degrees
Volar flexion, wrist	0-50 degrees	0-50 degrees	0-60 degrees
Metacarpophalangeal joint, small finger	0-50 degrees	0-85 degrees	0-90 degrees
Proximal interphalangeal joint, small finger	10-60 degrees	0-60 degrees	0-90 degrees
Distal interphalangeal joint, small finger	0-40 degrees	0-40 degrees	0-80 degrees

## Discussion

Atypical mycobacteria are opportunistic organisms that predominantly cause pulmonary disease and cutaneous lesions [2]. Infections of the musculoskeletal system by NTM are uncommon. In a retrospective analysis of more than 1000 patients, musculoskeletal involvement was present in approximately 3% of patients [4]. NTM have a predilection for the upper extremities, often causing flexor tenosynovitis in the hand and wrist [5,6]. While most cases of NTM tenosynovitis have been attributed to infection by *Mycobacterium kansasii* and *Mycobacterium marinum*, *Mycobacterium intracellulare* is emerging as an inciting organism [8]. NTM tenosynovitis primarily transmits through direct inoculation via trauma, surgical incisions, puncture wounds, or injections, but can also spread hematogenously from pulmonary disease in immunocompromised hosts [7]. In a retrospective analysis of 44 patients with culture-positive NTM infections of the upper extremity, 20 patients were immunosuppressed. These patients were more likely to present with systemic symptoms and had fewer known inoculation injuries [10]. In contrast, our patient presented with only localized symptoms and did not have a history of trauma to the affected site.

NTM tenosynovitis is a chronic and indolent disease, commonly presenting as painful swelling, erythema, or a palpable mass [10]. However, its nonspecific symptoms and radiographic features often lead to misdiagnosis, as they closely mimic other inflammatory and infectious conditions. Depending on the characteristics of the infection and its location, the differential diagnoses for NTM tenosynovitis include tuberculous tenosynovitis, rheumatoid arthritis, seronegative arthritis, synovial chondromatosis, and complex ganglion cyst, as well as *Vibrio vulnificus* or *Aeromonas hydrophila* infections [13-15]. The diagnosis of NTM tenosynovitis is supported by histopathological, microbiological, and radiographic findings.

Granulomatous inflammation, common in histopathological analysis, is a nonspecific finding recorded in up to 93% of NTM skin, soft tissue, and musculoskeletal infections [16]. While an acid-fast bacilli smear is more specific, it can result in negative results in over half of cases [10,16]. Diagnosis requires culture of the pathogen from the affected site, though repeated samples are sometimes necessary [11,12]. Positive synovial tissue culture is a reliable diagnostic method, and initial negative fluid cultures should not delay treatment. Rice body formation, while not pathognomonic, can suggest possible NTM infection. Unlike in rheumatoid arthritis, NTM-associated rice bodies typically surround the flexor tendon sheath rather than the bursae, and there is minimal bone or muscle involvement [17]. The primary utility of MRI is determining the extent of infection [10].

The diagnosis of NTM infections can be delayed due to insufficient clinical suspicion and limitations in bacterial detection methods. A study by Sotello et al. showed an average delay of four months in diagnosis, with some challenging cases experiencing diagnostic delays of up to 36 months. Those with longer diagnostic delays were shown to have worse clinical outcomes [10]. Patients experiencing longer diagnostic delays have been associated with worse clinical outcomes [10]. The management of NTM tenosynovitis typically requires a combination of surgical intervention and antibiotic therapy. The duration of treatment can vary, but at least six months of multi-drug antimycobacterial therapy based on the susceptibility of the isolated mycobacteria is recommended [18]. Despite the dual approach, some patients may require multiple debridements to control infection and prevent further complications [8]. In such cases, prolonged durations of antibiotic therapy up to a year or more may be necessary [19]. It has been suggested that immunocompromised status may contribute to treatment failure [5]. The patient in this case experienced significant improvement in symptomatology and hand functionality within four months postoperatively, with no evidence of disease progression.

## Conclusions

In this case report, we detail a rare instance of wrist and hand infection caused by *Mycobacterium intracellulare* in an immunocompromised patient, leading to flexor tenosynovitis. Our report underscores the significance of maintaining a high index of suspicion for NTM infection when assessing chronic hand swelling, particularly in individuals with compromised immune systems. Timely diagnosis is crucial in NTM tenosynovitis to prevent delays with potentially detrimental consequences. Due to its challenging nature, a comprehensive diagnostic approach incorporating acid-fast stains, cultures, histological examination, and imaging is recommended. While not pathognomonic, the presence of rice bodies can signal NTM infection, particularly in immunocompromised patients with chronic hand swelling after ruling out other etiologies. MRI plays a vital role in determining the extent of infection. Effective management necessitates prompt surgical intervention and referral to infectious disease specialists for appropriate selection of antibacterial therapy.
